# Burnout and its influencing factors between frontline nurses and nurses from other wards during the outbreak of Coronavirus Disease -COVID-19- in Iran

**DOI:** 10.17533/udea.iee.v38n2e03

**Published:** 2020-07-10

**Authors:** Tahere Sarboozi Hoseinabadi, Samaneh Kakhki, Gholamheidar Teimori, Somayyeh Nayyeri

**Affiliations:** 1 Nurse, M.Sc. Department of Nursing, School of Nursing and Midwifery, Torbat Heydariyeh University of Medical Sciences, Torbat Heydariyeh, Iran. Email: sarboozit1@thums.ac.ir Torbat Heydarieh University Torbat Heydariyeh University of Medical Sciences Iran sarboozit1@thums.ac.ir; 2 Pharmacist, Ph.D. Assistant Professor, Department of Nursing, School of Nursing and Midwifery, Torbat Heydariyeh University of Medical Sciences, Torbat Heydariyeh, Iran. Email: samane_ph@yahoo.com Torbat Heydarieh University Torbat Heydariyeh University of Medical Sciences Iran samane_ph@yahoo.com; 3 M.Sc. Department of Occupational Health Engineering, School of Health, Torbat Heydariyeh University of Medical Sciences, Torbat Heydariyeh, Iran. Email: teimorigh1@gmail.com Torbat Heydarieh University Torbat Heydariyeh University of Medical Sciences Iran teimorigh1@gmail.com; 4 Nurse, M.Sc. Department of Nursing, School of Nursing and Midwifery, Torbat Heydariyeh University of Medical Sciences, Torbat Heydariyeh. Iran. Email: s.nayyeri86@yahoo.com. Corresponding author. Torbat Heydarieh University Torbat Heydariyeh University of Medical Sciences Iran s.nayyeri86@yahoo.com; 5 Health Sciences Research Center, Torbat Heydariyeh University of Medical Sciences, Torbat Heydariyeh, Iran Torbat Heydarieh University Torbat Heydariyeh University of Medical Sciences Iran

**Keywords:** Burnout, psychological, nurses, coronavirus infections, risk factors, pandemics, cross-sectional studies., agotamiento psicológico, enfermeras y enfermeros, infecciones por coronavirus, factores de riesgo, pandemias, estudios transversales., esgotamento psicológico, enfermeiras e enfermeiros, infecções por coronavirus, fatores de risco, pandemias, estudos transversais.

## Abstract

**Objective.:**

To assess burnout level during an outbreak of COVID-19 and to identify influencing factors between frontline nurses and nurses from other wards.

**Methods.:**

This cross-sectional study makes comparison between two groups of nurses including frontline (exposure group) and other nurses working in usual wards (non-exposure group) in Torbat Heydariyeh city, Iran. Oldenburg Burnout Inventory (OLBI), Job stress questionnaire (JSQ), and questionnaires of hospital resources, family support, and measuring the fear of COVID-19 were used as research instruments.

**Results.:**

The scores of job stress and burnout in the exposure group with COVID-19 infection were significantly higher than in the non-exposure group (*p*=0.006 and *p*=0.002, respectively). Although, in univariate linear regression, employment status (*p*=0.047), experience in taking care of patient confirmed or suspected with COVID-19 infection (*p*=0.006), hospital resources (*p*=0.047), and job stress (*p*<0.001) were considered as significant risk factors for COVID-19-related burnout. In multivariate regression analysis, job stress (*p*=0.031, *β*=0.308) was considered as an only factor that has a significant relationship with COVID-19-related burnout.

**Conclusion.:**

The burnout level in frontline nurses was higher than other nurses, the most important influencing factor was the job stress. Regarding to negative effects of burnout on both physical and mental health nurses, it is suggested that a strong strategy be considered to reduce nurses' burnout to be able to control ongoing and future outbreaks successfully.

## Resumen

**Objetivo.:**

Evaluar el nivel de burnout durante un brote de COVID-19 e identificar sus factores influyentes entre las enfermeras de primera línea y enfermeras de otras salas.

**Métodos.:**

Este estudio transversal hace una comparación entre dos grupos de enfermeras: las de primera línea (grupo de exposición) y otras que trabajan en las salas habituales (grupo sin exposición) en la ciudad de Torbat Heydariyeh, Irán. El Inventario de Burnout de Oldenburg (OLBI), el cuestionario de estrés laboral (JSQ) y los cuestionarios de recursos hospitalarios, apoyo familiar y medición del miedo a COVID-19 se utilizaron para recolectar la información de la investigación.

**Resultados.:**

Las puntuaciones de estrés laboral y burnout en el grupo de exposición con infección por COVID-19 fueron significativamente más altas que en el grupo sin exposición (*p*=0006 y *p*=0.002, respectivamente). Aunque el análisis de regresión univariante, el estado de empleo (*p*=0.047), la experiencia en el cuidado de pacientes confirmados o sospechosos de infección por COVID-19 (*p*=0.006), los recursos hospitalarios (*p*=0.047) y el estrés laboral (*p*<0.001) se consideraron factores de riesgo significativos para el burnout relacionado con COVID-19; en el análisis de regresión multivariante, el estrés laboral (*p*=0.031, *β*=0.308) se consideró como un único factor que tiene una relación significativa con el agotamiento relacionado con COVID-19.

**Conclusión.:**

El nivel de agotamiento en las enfermeras de primera línea fue más alto que en el resto; el factor que más influye fue estrés laboral. Con respecto a los efectos negativos del burnout en su salud física y mental se sugiere considerar estrategias sólidas para reducirlo y poder controlar con éxito los brotes en curso y futuros.

## Resumo

**Objetivo.:**

Avaliar o nível de burnout durante um surto de COVID-19 e identificar seus fatores que influenciam entre enfermeiros de primeira linha e enfermeiros de outras enfermarias.

**Métodos.:**

Este estudo transversal compara dois grupos de enfermeiros: os da linha de frente (grupo de exposição) e outros que trabalham nas enfermarias habituais (grupo sem exposição) na cidade de Torbat Heydariyeh, Irã. Os questionários Inventário de Burnout de Oldenburg (OLBI), Job Stress Questionnaire (JSQ), Recursos Hospitalares, Apoio à Família e Medo de COVID-19 foram utilizados para coletar as informações da pesquisa.

**Resultados.:**

Os escores de estresse no trabalho e burnout no grupo de exposição á infecção por COVID-19 foram significativamente maiores do que no grupo sem exposição (*p*=0.006 e *p*=0,002, respectivamente). Apesar da análise de regressão univariada do status de emprego (*p*=0.047), experiência no atendimento a pacientes confirmados ou suspeitos de infecção por COVID-19 (*p*=0.006), recursos hospitalares (*p*=0.047) e estresse no trabalho (*p*<0.001) foram considerados fatores de risco significativos para burnout relacionados ao COVID-19. Na análise de regressão multivariada, o estresse no trabalho (*p*=0.031, *β*=0.308) foi considerado um fator único que possui uma relação significativa com a exaustão relacionada ao COVID-19.

**Conclusão.:**

O nível de exaustão nos enfermeiros de primeira linha foi maior que nos demais enfermeiros, o fator que mais influenciou foi o estresse no trabalho. Em relação aos efeitos negativos do burnout na saúde física e mental dos enfermeiros, sugere-se considerar estratégias sólidas para reduzi-lo e controlar com sucesso surtos no presente e no futuro.

## Introduction

The first known death from pneumonia caused by the virus named COVID-19 as a coronavirus family was reported in Wuhan, China, in December 2019.(1) In comparison with two other types of coronaviruses such as MERS and SARS, the present new coronavirus is spreading more quickly and has higher contagiousness, resulting pandemic that lead to international major health issue.(2) Due to the nature of new virus which is highly sensitive to mutation, there is no vaccine and specific antiviral treatment.(3) On the other hand, based on World Health Organization (WHO) report, generally, coronavirus are not only very stable at low and freezing temperature for a certain period, but also can persist on different surfaces for long time depending on temperature, humidity and light condition lead to global threats.(4) 

According to currently available information, older adults and people of any age who have serious underlying medical condition are at a higher risk of more serious COVID-19 illness and death.(1) Regarding the possible transmission modes of COVID-19 such as droplet and contact spread, considering both the preventive health plans (e.g. Society distancing, wearing the mask in public and frequent hand washing, etc.) and well-prepared hospitals are as essential factors in the event of pandemic.(5) From another point of view, healthcare workers especially frontline medical workers are faced with hectic, unpredictable events and urgent situation made them so frustrated especially when they have to face with unknown disease such as COVID-19 that cause work-related stress overtime.(6) Excessive workloads, lack of enough time for recovery and inadequate hospital facilities, on the other hand, are as important factors that put them under persistent pressure and result in job dissatisfaction and sometimes effect on the quality of patient care that leads to burnout gradually.(7) 

Job burnout is a syndrome resulting from chronic work-related stress, with symptoms characterized by emotional exhaustion including negative self-concept and inappropriate work attitude lead to lower productivity, impaired quality of work and a reduced interest in patients.(8) Due to the nature of nursing work experienced heavy workload, working long hours, night shifts and risk of exposure with infectious disease, the most studies on burnout were focused on nurses that sometimes remain as significant concerns, affecting not only work office but also individual life and sometimes can lead to increased risk of developing mental and physical health problems.(9) Since an outbreak of COVID-19 as an unknown disease that causes severe respiratory infectious disease and similar experience on SARS and MERS, nursing managers need to pay more attention to frontline nurses' burnout in association with their experiences of a nationwide MERS and SARS outbreak to be able to manage the conditions.(10) Thus, the aim of the present study is to assess and compared burnout level between frontline nurses and other nurses during an outbreak of COVID-19 and to identify influencing factors in order to provide basic information for lowering and preventing the level of burnout. 

## Methodology

### Study Participants and Setting

This cross-sectional study was conducted to determine the factors influencing on frontline nurses' burnout as an exposure group with experience in taking care of patients confirmed or suspected with COVID-19 infection in comparison to other nurses as non-exposure group in Torbat Heydariyeh city, Iran. Regards to our university decision to consider one hospital as a reception center and care for COVID-19 patients, the available nurses were estimated 266. So, 245 numbers of participants considering the inclusion and exclusion criteria were entered into the study (response rate 92%). According to hospital policies, frontline nurses as an exposure group have to work 12 hour-rotating shifts for one week and have a week off, while other nurses as a non-exposure group entered in the study have the same working condition as before. Moreover, all frontline nurses have to use personal protective equipment (PPE). Inclusion criteria in the study were all nurses working in target hospital, and exclusion criteria were lack of willingness to participate in the study and pregnant participants. Data were collected from March 10 to April 3, 2020 during the outbreak of COVID-19 in our city.

### Ethics Approval

This study was approved by the ethics committee of Torbat Heydariyeh University of Medical Sciences. All participants fulfilled the informed written consent.

### Measuring tools

Regards to literature review, we considered six essential components including burnout questionnaire and 5 various items as influencing factors on burnout such as hospital resources, family support, occupational stress, demographic information, and measuring the fear of COVID-19. The Persian version of burnout and job stress questionnaires was used in this study. The questionnaires of hospital resources, family support, and measuring the fear of COVID-19 translated into Persian language and the content validity of the measurement tools were assessed by 3 nursing specialists. The developed tools were also translated into Persian and then translated back into English. To analyze its validity, Small-case study with 30 healthcare workers was conducted before starting the main trial.

The *Oldenburg Burnout Inventory* (OLBI) developed by Demerouti *et al* was used in the present study to measure burnout,(11) which consists of 16 items; eight of questions are about emotional exhaustion and job dissatisfaction. The scale ranges from 1 (*strongly disagree*) to 5 (*strongly agree*) that high score means high level of burnout. In fact, The Oldenburg Burnout Inventory (OLBI) has a high validity and reliability and It has been translated into several different languages around the world.(12) The psychometric characteristics of the Persian version of the OLBI were confirmed by Larki *et al*. through confirmatory factor analysis and estimation of internal consistency coefficients. Also Larki *et al*. declared 0.77 Cronbach’s alpha coefficient.(13)

*Job Stress Questionnaire* (JSQ) developed by Parker and Decotiis was first used to determine the cause of organizational stress. (14) The JSQ is considered to assess the anxiety stress and time stress. This scale consists of 12 items scored on a 5-point scale ranging from one extreme to other (*strongly disagree* to *strongly agree*), with these categories scored from one to five. A high score means a high level of stress. Parker and Decotiis(14) reported the reliability of the two dimensions of this scale using Cronbach's alpha for the time pressure dimension of 0.86 and for the job-related anxiety dimension 0.74. In the study of Iraji and Sohrabi,(15) the reliability coefficients of the questionnaire were obtained using Cronbach's alpha coefficient for time pressure of 0.83, for anxiety dimension 0.82, and its overall reliability and validity coefficient as 0.90 and 0.68, respectively. The scales to measure the hospital resource and family support level were used based on previous studies.(16,17) The scales for hospital resources including three items was ranged from 1(*strongly disagree*) to 4 (*strongly agree*) which means the more satisfaction of hospital resources for the treatment of COVID-19 lead to the higher score. According to Kim *et al.* study the Cronbach's α was considered 0.81. In our study, Content validity index was 0.78.

Family and friend supports scale including four items was ranged from 1 (strongly disagree) to 4 (strongly agree) with a high score meaning high support.(10,18) Cronbach’s α of the scale in previous study was done by Kim *et al.* was 0.80. In our study, Content validity index was 0.75. Eventually, the last item as an influencing factor was measuring the fear of COVID-19 taking from previous study as mentioned before. It was a ten-point question, with higher score reflecting the greater fear of COVID-19 infection.(17)

### Statistical analysis

Data analysis were done using SPSS version 21. Quantitative variables were stated as mean ± standard deviation/SD and categorical variables summed-up as number (percentage). The socio-demographic characteristics of participants between the two groups were assessed and compared using the chi-square test and Fishers exact test. The independent sample t-test was used for non-categorical variables (age, work experience, and characteristics of work-related variables). Univariate logistic regression was performed to determine the influencing factors for COVID-19-related burnout. Significant variables were selected for multiple regression analysis to further explore this association. Inferences was based on β and *p*-values; in all analyses, level of significance was defined *p*<0.05.

## Results

Of all the 245 participants, 151 (61.63%) were included in exposure group to COVID-19 infection and 94 (38.37%) in non-exposure group to COVID-19 infection. The mean age of participants in exposure and non-exposure group were 31.9 (range 23-54) and 31.6 (range 22-54) years old respectively. The mean work experience of subjects in the exposure and non-exposure groups were 6.93±5.69 (1-25) and 6.25±6.24 (1-29) years, respectively. The demographic characteristics including age, gender, marital status, work experience, qualification, and history of underlying disease were similar between the two groups (Table 1). Statistically, significant differences were just found between the two groups for the following characteristic: the employment status of individuals (*p*<0.001). Actually, based on statistical analysis, the exposure group had bachelor degrees (*n*=125, 82.8% vs. *n*=73, 77.7%) more often than the non-exposure group; while they had lower associate’s degree (n=26, 17.2% vs. n=21, 22.3%) in comparison with the non-exposure group. 

In terms of employment statue, the percentage of participants with contracted and corporate recruitment were (*n*=54, 35.8%), (n=45, 29.8%) respectively and higher than non-exposure group (*n*=10, 10.6% vs. *n*= 25, 26.6). On the other hands, the percentage of participants with official recruitment (*n*=17, 11.3% vs. n=29, 30.9%) and temporary job (*n*=35, 23.2% vs. *n*=30, 31.9%) was lower in the exposure group in compared with the non-exposure group (Table 1).

**Table 1:** Socio-demographic characteristics of the respondents by group

Variable	**Exposure to COVID-19 (*n*=151)**	Non-exposure to COVID-19 (*n*=94)	***t* or *X*** ^*2*^	***p*-value**
Age in years; Mean±SD	31.9± 6.5	31.6 ±7.4	-0.458	0.647
Gender; *n* (%)			0.960	0.327
Male	82 (54.3)	45 (52.1)		
Female	69 (45.7)	49 (47.9)		
Marital Status; *n* (%)			2.230	0.135
Single	47 (31.1)	21 (22.3)		
Married	104 (68.9)	73 (77.7)		
Work experience in years; Mean±SD	6.93±5.7	6.25±6.2	-0.864	0.388
Qualification; *n* (%)			0.980	0.322
Associate’s degree	26 (17.2)	21 (22.3)		
Bachelor	125 (82.8)	73 (77.7)		
Employment status; *n* (%)			27.718	<0.001
Temporary	35 (23.2)	30 (31.9)		
Corporate	45 (29.8)	25 (26.6)		
Contracted	54 (35.8)	10 (10.6)		
Official	17 (11.3)	29 (30.9)		
History of underlying disease; *n* (%)	15 (9.9)	17 (18.1)	3.390	0.066
History of hospitalization; *n* (%)	27 (17.9)	21 (22.3)	0.731	0.392

The total mean score of family and friends support was 2.63 out of 5 [2.60 (*n*=151) in the exposure group and 2.68 (*n*=94) in non-exposure group], the total mean score for hospital resources for treatment was 2.08 out of 5 (2.04 vs. 2.14), COVID-19 related job stress was 3.07 out of 5 (3.22 vs. 2.85) and the fear of COVID-19 as a 10-point question was also assessed and the mean score was 6.08 out of 10 (6.29 vs. 5.75). The mean value of COVID-19-related burnout scores for both groups was 2.57 out of 5 [2.61±0.27 (*n*=151) in exposure group and 2.51±0.23 (*n*=94) in non-exposure group]. As a result, statistically, job stress and burnout were the variables that makes the significant difference in comparison with other variables (*p*=0.006 and *p*=0.002); whereas there were no significant differences between the variables such as fear of COVID-19 infection, hospital resources, and family support in the two groups. 

**Table 2:** Comparing the characteristics of work-related variables in the two groups

Variable	**Exposure to COVID-19 (*n*=151)**	Non-exposure to COVID-19 (*n*=94)	*t*	***p*-value**
Support from family and friends	2.6±0.4	2.7±0.4	1.57	0.117
Hospital resources for treatment of COVID-19	2.0±0.6	2.1±0.6	1.28	0.202
Fear of COVID-19 infection	6.3±2.9	5.7±2.7	-1.44	0.150
COVID-19 related job stress	3.2±0.9	2.8±0.7	-3.20	0.002
COVID-19 related burnout	2.6±0.2	2.5±0.2	-2.86	0.006

In this study, univariate and multivariate linear regression analyses were conducted. Summary of variables associated with influencing factors for COVID-19-related burnout in 245 participants are described in Table 3. To summarize, employment type (*p*=0.047), experience in taking care of patients with COVID-19 infection (*p*=0.006), hospital resources (*p*=0.047), and job stress (*p*<0.001) were considered as significant risk factors for COVID-19-related burnout in univariate analysis. Furthermore, we found that COVID-19 related job stress (*p*=0.0314, *β*=0.308) is also significant variable in the multivariate regression analysis.

**Table3:** Analysis of variables associated with influencing factors for COVID-19- related burnout

Variables	Univariate analysis	Univariate analysis	Multivariate analysis	Multivariate analysis
Variables	Beta (95% CI)	***p*-value**	Beta	***p*-value**
Age	0.020 (-0.004-0.006)	0.752		
Gender	0.043 (-0.045-0.090)	0.506		
Marital status	0.056 (-0.042-0.108)	0.386		
Work experience	0.033 (-0.004-0.007)	0.608		
Qualification	0.032 (-0.096-0.159)	0.623		
Employment type	0.128 (0.00-0.034)	0.047	0.087	0.152
History of underlying disease	0.007 (-0.094-0.105)	0.914		
History of hospitalization	-0.026 (-0.102-0.067)	0.686		
Experience in caring for COVID-19 infection	0.175 (0.027-0.164)	0.006	0.113	0.067
Support from family & friends	0.028 (-0.065-0.102)	0.668		
Hospital resources for treatment of COVID-19	-0.128 (-0.001-1.00)	0.047	-0.052	0.402
Fear of COVID-19 infection	0.098 (-0.003-0.021)	0.128		
COVID-19 related job stress	0.350 (0.068-0.138)	<0.001	0.308	<0.001

## Discussion

Health care workers, especially nurses, are among those who are directly exposed to patient diagnosed with various and sometimes unknown disease cause stress and emotional exhaustion lead to burnout gradually. Regards to new studies demonstrated the pivotal role of nurses in infection prevention, infection control, and public health.(19) So, considering physical and mental health are of particular importance and identifying the source of stress in order to improve the situation and reducing job risks. The main goal of the present study was to assess and compare burnout and its influencing factors such as hospital resources, family support, job stress, demographic characteristics and measuring the fear of COVID-19 between frontline nurses taking care of patients confirmed or suspected with COVID-19 infection and other nurses working in usual wards.

Based on statistical results, demographic characteristics including age, gender, marital status, and work experience were the same in both groups, also variables of hospital resource, family support, and fear of COVID-19 didn't show a significant difference between two groups of exposure and non-exposure group; while job stress and burnout level in exposure group were higher than non-exposure group. Nurses who were tasked caring for patients with coronavirus infection, had long work shifts; while other nurses had three-shift system. Actually, long work shifts (20,21) and emotional stress such as away from family.(22) May be one reason for higher level of burnout in exposure group in comparison with non-exposure group in our study. So, physical and mental fatigue, stress and anxiety, and burnout among frontline nurses in comparison with other nurses can result from a range of factors such as resource limitations, longer shifts, and sleep disorders.(23) In contrast of our findings, the results of study by Wu *et al*., demonstrated frontline medical staffs working on the COVID-19 experienced lower levels of burnout than healthcare workers working on usual wards, and it might be because they felt the situation under control,(24) while another study was done by Kim and colleagues for assessing burnout among Emergency department (ED) nurses taking care patients with MERS-CoV infection reported higher burnout than nurses in other hospital departments, which was consistent with the results of our study.(10)

The results of univariate linear regression showed that variables such as employment statue; work experience, hospital resource, and job stress were known as influencing factors on burnout. Furthermore, based on our finding, sustainable employment condition and strong hospital resource can reduce burnout. Although, regards to study by Kim *et al*. reported poor hospital support, hospital resources was significantly higher than our study.(10) 

The results of multivariate regression analysis showed job stress as the main related factor with burnout. Based on our result, the main factor among all influencing criteria on burnout is job stress; the second and third influencing factors were hospital resources and family and friend support respectively. However, some systematic review has been reported the hospital resources and family support as major influencing factors for nurses' burnout.(23,25,26)

To cope with such though situation, it is strongly suggested to be well- prepared as hospital facilities, staffs and developing systematic infection-control guidelines. On the other hand, based on previous similar studies and our findings focused on frontline staffs during outbreaks, nursing managers need to pay much more attention to health care worker and design the long-term program such as preventive plans, reducing shift work, and find the sources of stress and resolve them to manage not only the ongoing outbreak, but we will also be able to control the future pandemic. 

One limitation of this study is small number of sample size, bias in filling out the questionnaire, and not considering selecting the participants from other region of country. In addition, job attitude, having the previous experience with contagious disease is better to added for future study. Due to prolonged involvement of people with COVID-19, we also suggest doing this study in a longer period of time during the COVID-19 outbreak to compare the present results with future data to achieve the better conclusion. However, this is the first study of its kind for evaluating nurses' burnout.

## Conclusion

Due to the nature of nurses' job faced with various diseases and sometimes unknown cause much more stress lead to burnout overtime and based on the results, it is confirmed the burnout level is almost higher in frontline nurses in comparison to other nurses. On the other hand, numerous evidences illustrated the strong relationship between burnout and problems influenced both work and individual life resulted in uncontrolled outbreak. So, in order to lower the level of burnout, nursing managers must make more efforts to reduce job stress and burnout through finding stress sources and resolving them. It is highly recommended to develop effective and systematic burnout management programs to be able to cope against possible future outbreaks of infectious disease may lead to pandemic.
